# Cognitive training based on functional near-infrared spectroscopy neurofeedback for the elderly with mild cognitive impairment: a preliminary study

**DOI:** 10.3389/fnagi.2023.1168815

**Published:** 2023-07-26

**Authors:** Ilju Lee, Dohyun Kim, Sehwan Kim, Hee Jung Kim, Un Sun Chung, Jung Jae Lee

**Affiliations:** ^1^Department of Psychology, College of Health Science, Dankook University, Cheonan, Republic of Korea; ^2^Department of Psychiatry, Dankook University Hospital, Cheonan, Republic of Korea; ^3^Department of Psychiatry, College of Medicine, Dankook University, Cheonan, Republic of Korea; ^4^Department of Biomedical Engineering, College of Medicine, Dankook University, Cheonan, Republic of Korea; ^5^Department of Physiology, College of Medicine, Dankook University, Cheonan, Republic of Korea; ^6^Department of Psychiatry, School of Medicine, Kyungpook National University, Daegu, Republic of Korea

**Keywords:** functional near-infrared spectroscopy, neurofeedback, elderly, cognitive training, cognitive impairment

## Abstract

**Introduction:**

Mild cognitive impairment (MCI) is often described as an intermediate stage of the normal cognitive decline associated with aging and dementia. There is a growing interest in various non-pharmacological interventions for MCI to delay the onset and inhibit the progressive deterioration of daily life functions. Previous studies suggest that cognitive training (CT) contributes to the restoration of working memory and that the brain-computer-interface technique can be applied to elicit a more effective treatment response. However, these techniques have certain limitations. Thus, in this preliminary study, we applied the neurofeedback paradigm during CT to increase the working memory function of patients with MCI.

**Methods:**

Near-infrared spectroscopy (NIRS) was used to provide neurofeedback by measuring the changes in oxygenated hemoglobin in the prefrontal cortex. Thirteen elderly MCI patients who received CT-neurofeedback sessions four times on the left dorsolateral prefrontal cortex (dlPFC) once a week were recruited as participants.

**Results:**

Compared with pre-intervention, the activity of the targeted brain region increased when the participants first engaged in the training; after 4 weeks of training, oxygen saturation was significantly decreased in the left dlPFC. The participants demonstrated significantly improved working memory compared with pre-intervention and decreased activity significantly correlated with improved cognitive performance.

**Conclusion:**

Our results suggest that the applications for evaluating brain-computer interfaces can aid in elucidation of the subjective mental workload that may create additional or decreased task workloads due to CT.

## 1. Introduction

Dementia is a significant concern in Korea which has the highest rate of aging among the Organization for Economic Co-operation and Development nations and is projected to have the greatest number of older individuals in 2075. In 2018, approximately 750,000 elderly Koreans were diagnosed with dementia; 75% of the cases were caused by Alzheimer’s. As the population ages, the prevalence of dementia in Korea is expected to increase to three million by 2050, and approximately 1.7 million Koreans are estimated to live with mild cognitive impairment (MCI) (Nam et al., 2019).

Despite the heterogeneity of the clinical features and causes, MCI is considered an intermediate stage between normal aging and dementia, and approximately 12–41% of older adults are at risk of developing dementia (Wenisch et al., 2007). Managing MCI is essential because it may indicate the earliest manifestation of neurodegenerative diseases with progressive deterioration in daily life function (Albert et al., 2011; Hernes et al., 2021). Given the increasing prevalence of MCI and early-stage dementia, numerous non-pharmacological interventions have been examined to delay the onset and mitigate the progressive decline in daily functional abilities associated with these conditions (Greenaway et al., 2013; Lee and Kim, 2013; Jeong et al., 2014).

Cognitive training (CT), specifically designed to improve core cognitive functions such as working memory, attention, and executive function, is used in the clinical field with the restorative paradigm. In particular, patients with MCI or early-stage dementia show a working memory deficit, which can be aided by corresponding changes in several neuroplastic mechanisms in the brain (Galante et al., 2007). Previous studies have shown that CT has a significant effect on improving memory and cognitive function in patients with MCI and dementia (Jang et al., 2012; Lee and Kim, 2013); computer-based CT also contributes to the improvement of memory and attention (Barnes et al., 2009). Numerous studies have suggested the benefits of cognitive enhancement with CT; however, these programs have limitations, such as the lack of objective indicators of whether training progression is effective in real-time, and it is also difficult to set individualized training difficulty for MCI or dementia patients.

Neurofeedback is a promising non-invasive neuromodulation tool that can change neural activity with real-time feedback. The body of evidence implies that learning to control the activation of specific brain regions is related to improving cognitive functions and behavior (Sulzer et al., 2013; Acevedo et al., 2022). Some studies have reported the benefits of neurofeedback for attention deficit hyperactivity disorder (Lofthouse et al., 2012; Thibault et al., 2018), depressive disorder (Choi et al., 2011; Lee et al., 2019), post-traumatic stress disorder (Nicholson et al., 2020; Panisch and Hai, 2020), and MCI (Lavy et al., 2021; Acevedo et al., 2022; see review Trambaiolli et al., 2021).

In this study, we developed a CT program to increase the effectiveness of brain function in patients with MCI by combining CT and neurofeedback systems. In particular, we provided real-time feedback based on brain activity to the participants regarding their performance on CT. Near-infrared spectroscopy (NIRS) has provided neurofeedback by measuring changes in oxygenated hemoglobin in the prefrontal cortex (PFC) (Quaresima and Ferrari, 2019). Due to the advantages of feasibility and relatively lower cost, fNIRS-neurofeedback could present a suitable and potent alternative to EEG and functional magnetic resonance imaging (fMRI) neurofeedback, demonstrating significant potential for clinical transference in the realm of neurofeedback (Kohl et al., 2020). Studies have shown the advantages of providing neurofeedback via a CT program (Hosseini et al., 2016; Acevedo et al., 2022). The researchers suggested that neurofeedback utilizing CT has the potential to be more engaging and reinforcing than the classical restoration paradigm for participants, as it allows real-time monitoring of brain activity during training. By actively monitoring their brain signals, participants may be more motivated to engage in training and experience greater accomplishment and progress (Hosseini et al., 2016). Functional NIRS (fNIRS)-based neurofeedback has recently been studied; however, there is a lack of research on elderly or MCI patients. To the best of our knowledge, only one study has tested the effectiveness of combining CT and fNIRS-based neurofeedback in an elderly population (Acevedo et al., 2022). In addition, to increase the training effect of neurofeedback and apply it to daily life, an experimental design combining virtual reality (VR) and neurofeedback training has also been proposed (Hudak et al., 2017). Given this evidence, the utilization of VR in conjunction with CT and fNIRS-neurofeedback represents a novel method to address the cognitive deficiencies of geriatric patients with MCI. The fully immersive and interactive characteristics of VR-based training modalities can enhance patients’ engagement and motivation during the training process, while the incorporation of fNIRS offers valuable insight into the neural underpinnings of cognitive enhancement. The results of this study have potential to expand the efficacy of CT for MCI patients and further understanding of the neural mechanisms underlying cognitive deterioration.

## 2. Materials and methods

The study protocol and neurofeedback procedure in this research were designed in accordance with the essential criteria outlined by the CRED-nf (Consensus on the Reporting and Experimental Design of clinical and cognitive-behavioral neurofeedback studies) to ensure reproducibility and facilitate a structured evaluation of the investigation (provided in Supplementary material; Ros et al., 2020).

### 2.1. Participants

Participants were recruited via advertisements and posters, and from a local dementia center in the Chungcheongnam-do Province, Republic of Korea. This study was approved by the Dankook University Hospital Review Board (IRB no. 2021-06-012). Participants were provided information about the study, including the eligibility criteria and procedures; written informed consent was obtained from all participants. The exclusion criteria were as follows: (1) any history of epilepsy or brain surgery, (2) patients diagnosed with neurologic or psychiatric disorders that could affect cognitive function, and (3) patients who had participated in any other study to improve their cognitive function. The diagnosis of MCI was established by a qualified psychiatrist, who carefully reviewed the results of a comprehensive neuropsychological evaluation conducted by an experienced clinical psychologist. MCI was diagnosed according to the consensus criteria from the International Working Group on MCI. The presence of objective cognitive impairment was ascertained when the performance of the participants was −1.5 SD or below the age-, sex-, and education-adjusted norms in any of the neuropsychological tests (Petersen, 2004).

In this preliminary study, our heuristic approach guided us to aim for a minimum of 40 participants, establishing a foundational dataset for our analysis (Lakens, 2022). However, our study faced the unforeseen challenge of participant dropout due to the COVID-19 pandemic mandated that we proceed with our research using a diminished final sample size for our analysis.

Fifteen elderly patients with MCI {12 females, mean [standard deviation (SD)] age = 70.92 (5.86)} participated in this study. One patient refused to participate after the baseline measurement, and one patient’s data could not be used for the study results owing to the malfunction of the NIRS device. Finally, data from 13 patients were analyzed (provided in the format of a CONSORT diagram).

### 2.2. Clinical assessments

We used a set of cognitive tests to measure the effects of VR + CT neurofeedback on executive function. We used Oculus Quest 2 VR headset (Meta Platforms, Inc.). All participants performed the following tests at baseline and 4 weeks after the end of the training protocol.

#### 2.2.1. Consortium to establish a registry for Alzheimer’s disease – neuropsychology battery

Each participant was examined by clinical psychologists with advanced training in neuropsychiatry and dementia, according to the protocol of the Korean version of the consortium to establish a registry for Alzheimer’s disease (CERAD) clinical assessment battery (Lee et al., 2002). An experienced psychiatrist performed eight subtests from the CERAD-NP battery. These subtests consisted of Verbal fluency, the Boston naming test, the Korean mini-mental state examination, word list memory, constructional praxis, word list recall, word list recognition, and constructional recall. The administration procedures have been described in detail (Lee et al., 2004).

#### 2.2.2. Digit span test

The digit span test is a neuropsychological test most commonly used to measure attention/working memory. It has been widely used to assess patients with degenerative disorders because it is easy to perform (Kang et al., 2002). The digit span test consists of digit span forward and backward. Three scores, including digit span forward and backward, and the total scores were used in this study.

#### 2.2.3. Frontal assessment battery

The frontal assessment battery (FAB) was developed to test executive function. It is a short cognitive and behavioral battery that evaluates six domains of frontal lobe function: conceptualization, mental flexibility, motor programming, sensitivity to interference, inhibitory control, and environmental autonomy (Dubois et al., 2000). The battery determines the reliability and validity of the Korean version of the FAB (K-FAB) among elderly patients (Kim et al., 2010).

#### 2.2.4. n-Back test

In our study, we decided to implement the n-back test in a specific manner. Informed by previous research indicating the quantification of cognitive task workload (Owen et al., 2005; Herff et al., 2013), the two-back test was chosen to measure improvements in working memory performance. In this test, participants were required to respond when a stimulus matched the one presented two steps earlier. Participants pressed the handheld controller button of the VR device upon recognizing a target.

Considering our study population of individuals with MCI, we determined the two-back task to be most appropriate. It presented an optimal challenge to effectively engage working memory while still being attainable for our participants (Belleville et al., 2014). Consequently, the two-back task was utilized as the primary outcome measure in our study. The analysis focused on reaction time and the number of correct responses, ensuring the robustness of our results.

### 2.3. fNIRS data acquisition

Functional NIRS records changes in oxygenated (O_2_Hb) and deoxygenated (HHb) hemoglobin relative to a baseline, and the amount of local O_2_Hb infers the amount of local brain activation via the process of hemodynamic coupling, wherein increases in cortical activation lead to increases in O_2_Hb and decrease in HHb (Haeussinger et al., 2014). While most commercial NIRS systems only measure changes from a baseline, our study utilized the ISS OxiplexTS (ISS, USA), a commercially available frequency-domain NIRS (fdNIRS) system that can calculate absolute hemoglobin concentrations (Fantini et al., 1999). In this fdNIRS, unlike continuous-wave-based fNIRS, the short separation channel is not essential for effectively reducing noise in the signal of the long channel. The probe includes one detector and four fiber optic sources, emitting light at 690 and 830 nm into the tissue with a modulated frequency of 110 MHz. After a 30-min warm-up period and calibration with phantoms of known absorption and scattering coefficients, the NIRS probe was placed on the right forehead as close to the hairline as possible and secured with Velcro straps. The frequency domain-multiple distance NIRS (ISS, USA) was used for pre-and post-tests and neurofeedback sessions. The optode sensor featured a flexible rubber molded construction with four emitter positions with emitter-detector distances of 2.0, 2.5, 3.0, and 3.5 cm. The optode sensor was placed at the center with the innermost channel of the international 10–20 electroencephalogram system (Jasper, 1958).

### 2.4. Neurofeedback procedure

We conducted a delayed working memory task to proceed with the neurofeedback training. Reflecting on the characteristics of older adults in that they may not have known letters, we provided images that can be encountered in daily life as memory tasks. In each trial, a set of five varied images was presented, chosen randomly from a pool of 80 unique images representing a wide range of everyday items, from animals to household goods to clothing. Once an image was correctly identified during a trial, it was removed from the selection pool for subsequent trials, ensuring that the stimuli remained fresh and engaging for the participants throughout the neurofeedback training.

**FIGURE 1:**
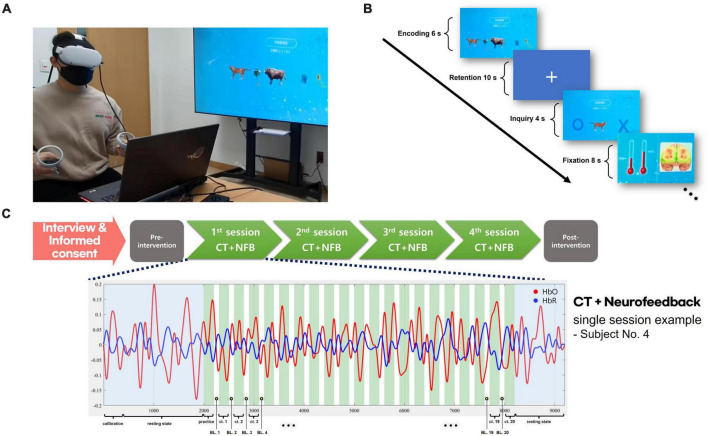
VR + CT-neurofeedback procedure. **(A)** A demonstration of conducting VR + CT-neurofeedback. **(B)** Cognitive training timeline with neurofeedback. In each trial, a 6-s encoding phase presented an image set, followed by a 10-s retention phase, and a 4-s inquiry phase to identify the initial stimuli. An 8-s fixation period separated trials. Participants were briefed that variations in the bar graph signified alterations in their brain activity. During the fixation phase, changes in fNIRS values from the previous training trial were displayed as a bar graph (red thermometer); the baseline is the left bar graph and the result of the previous trial is the opposite. **(C)** Overview of the VR + CT-neurofeedback process. Following the completion of an initial interview and informed consent, participants undergo clinical assessments. Subsequently, they attend the hospital for VR + CT-neurofeedback sessions once weekly. After completing four such training sessions in total (total 4 weeks), post-intervention clinical assessments are performed identically to the initial ones. Each session consists of 20 CT trials, and a practice trial is conducted before the commencement of actual training.

In the procedure of the CT combined with neurofeedback, the image set was presented to the participant for 6 s as the encoding phase in each trial. A delay period of 10 s was used as the retention phase, following a single image presented on the VR screen. In the inquiry phase, the participant had to respond within 4 s if the image was included in the original stimuli set. The fixation period was 8 s to separate the phase from the subsequent trial. Each training session consisted of 20 trials, and the participants underwent once-weekly training sessions for 4 weeks (see Figure 1).

The brain region selected for neurofeedback training was determined based on previous evidence. Several studies have found that deficits in working memory and executive functions are the most common symptoms in patients with MCI (Albert et al., 2001; Huntley and Howard, 2010; Aurtenetxe et al., 2016). Working memory is highly associated with the dorsolateral prefrontal cortex (dlPFC) and significant left-hemisphere lateralization in the PFC for delayed working memory tasks has shown different activation based on manipulation of the cognitive load (i.e., information size or temporal lag). Given this evidence, we chose the left-dlPFC as the training center for this experiment.

Within our study design, our objective was indeed to enhance the neural activity of the participants during the tasks. Consistent with research showing that cognitive task loading often results in increased frontal lobe oxyhemoglobin (OHb) activity, the threshold for this augmentation was set at a 20% increase from either the baseline neural activity or the recorded activity from the previous trial. The threshold was not a static value but was adaptively updated after each trial. Given the dynamic nature of neural activity, our approach ensured that the threshold for each trial was individualized and reflected the participant’s most recent performance.

Before initiating the sessions, participants were informed that the variations (increase or decrease) in the bar-graph during the neurofeedback training were representative of changes in their brain activity. In each trial, the participants were presented with feedback regarding brain activity in the left-dlPFC and their behavioral performance. The NIRS signal was recorded during the trial (i.e., fixation, encoding, and retention phases), and OHb measurements over a 24-s window were used for neurofeedback. The feedback was visually presented as a bar plot that showed changes in neural activity during the previous trial. Absolute OHb values were calculated to represent decreased or increased activity in the targeted channel.

### 2.5. fNIRS data analysis

Structured preprocessing for fNIRS data was performed to analyze pre-post n-back task-based NIRS data and NIRS-neurofeedback training data. The MATLAB toolbox, NIRS-KIT, was used for data preparation, quality control, preprocessing, and statistical analyses (Hou et al., 2021). Raw signals were bandpass-filtered between 0.01 and 0.08 Hz using a third-order IIR Butterworth filter to eliminate physiological artifacts. The NIRS-KIT also provides motion correction to effectively remove both spike artifacts and baseline shifts, and temporal derivative distribution repair was chosen (Fishburn et al., 2019).

Next, an individual-level analysis was conducted to obtain task-related neural activity based on the general linear model (GLM). The estimation of the GLM parameters from the task variables was used to calculate the weight coefficient in the linear model after inputting the user-defined contrast vectors with condition-wise effects of interest. The block average amplitude value can be estimated using the above procedure for the group-level analysis. Finally, we used the EasyTopo toolbox, which provides interpolated three-dimensional visualization on a standard brain surface to present three-dimensional visualization (Tian et al., 2013). The integrated statistical values and the Montreal Neurological Institute and Hospital coordinates of each channel were calculated, and all colors of the brain surface represented the *t*-statistic values of the group-level *t*-test.

### 2.6. Statistical analysis

All statistical analyses were performed using the R statistical software version 4.2.1 (R Foundation for Statistical Computing, Vienna, Austria). Descriptive statistics were used to describe the demographic and clinical characteristics of the participants and cognitive performance. The clinical assessments and behavioral performance data were summarized as mean and SD for quantitative variables, while block-averaged fNIRS data were expressed as the mean and standard error of the mean.

As our study did not include a SHAM or control group, we used a paired *t*-test to evaluate the statistical significance of pre-post changes in neuropsychological assessments, behavioral performance, and neural activation (i.e., O_2_Hb) in the two-back task. Bonferroni corrections to control family-wise error rates were used to assess statistical significance in the context of multiple tests. In cases where the assumption of normality was violated, we used two-tailed Mann–Whitney U or Wilcoxon signed-rank tests.

Additionally, Pearson’s correlation analysis was performed to identify the association between behavioral effects and neural activation induced by neurofeedback training. Correlation analysis was also conducted to test whether CT in parallel with fNIRS neurofeedback contributed to improving working memory.

An additional measure used in our study, termed “Delta Activation (Δ activation),” was derived from task-related neural activity (indicated by the beta coefficients from the GLM). This measure was calculated as the difference between neural activity during the post-intervention and pre-intervention sessions. We also calculated Δ%, which provided us with a sense of the relative change in neural activity, expressed as a percentage. This measure provides a useful way to quantify changes in neural activation due to the intervention.

## 3. Results

### 3.1. Demographics and clinical assessments

Of the 15 outpatients enrolled, one patient did not show up for the assigned sessions after the baseline assessment and another patient’s data were excluded because of a device malfunction. The following results are based on the final participant set consisting of 13 participants in the experiment. They successfully underwent baseline and post-intervention evaluations and completed all VR + CT neurofeedback training sessions.

Participants’ mean age was 70.92 years (SD = 5.86), and there was only one male participant. The results showed changes in neuropsychological and cognitive measures for pre-and post-VR + CT neurofeedback sessions (Table 1). Significant improvement in the working memory scores was observed after the 4-week training compared to pre-intervention: the Boston naming test (*paired*-*t*_[12]_ = 2.22; *p* = 0.023), word list recognition (*paired*-*t*_[12]_ = 2.12; *p* = 0.027), constructional recall (*paired*-*t*_[12]_ = 2.29; *p* = 0.020), and digit span test (forward: *paired*-*t*_[12]_ = 2.01; *p* = 0.032, total: *paired*-*t*_[12]_ = 3.24; *p* = 0.003). There was a similar improvement in the working memory task: the two-back task was performed accurately (*paired*-*t*_[12]_ = 2.25; *p* = 0.021).

**TABLE 1:** Demographics and clinical assessments.

	Pre-VR + CT-neurofeedback		Post-VR + CT-neurofeedback			
Variable	(*n* = 13)		(*n* = 13)			
	Mean	SD	Mean	SD	df	*T*-value
**Demographics**						
Age	70.92	5.86				
Sex (F/M)	12/1					
**CERAD-NP**						
Verbal fluency	15.23	3.42	15.31	3.12	12	0.06
Boston naming rest	12.15	2.51	13.08	1.26	12	2.22*
MMSE	26.69	2.84	26.31	2.56	12	−0.67
Word list memory	18.69	3.09	19.46	3.20	12	1.19
Constructional praxis	10.23	1.01	10.77	0.44	12	1.72
Word list recall	6.15	1.41	6.46	1.33	12	0.88
Word list recognition	8.62	1.45	9.23	1.30	12	2.12*
Constructional recall	6.62	3.40	8.08	2.96	12	2.29*
**Digit span test**						
Forward score	7.23	2.27	8.76	1.78	12	2.01*
Backward score	4.00	1.63	4.81	1.96	12	1.74
Total score	11.23	2.52	13.54	2.88	12	3.24**
**Frontal assessment battery**						
Total score	15.23	2.35	16.00	1.73	12	1.30
n-Back task						
Reaction time	3.67	0.56	3.88	0.62	12	1.75
Correct	18.46	6.72	22.54	3.80	12	2.25*
Accuracy (%)	68.37%		83.47%			

Bold text signifies significant differences between variables. *p < 0.05, **p < 0.01.

VR, virtual reality; CT, cognitive training; SD, standard deviation; MMSE, mini mental state examination.

### 3.2. fNIRS data

**FIGURE 2:**
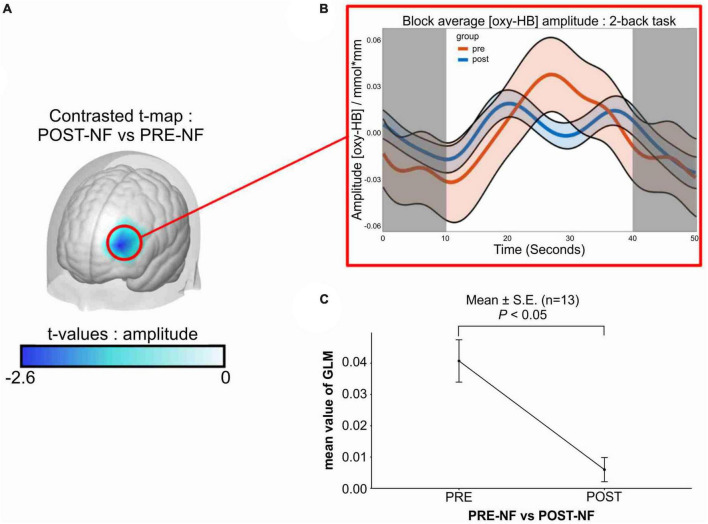
Changes in left-dlPFC activity after VR + CT-neurofeedback. **(A)** Difference between pre-and post-intervention in left-dlPFC activity in response to the training: contrasted t-map shows significantly decreased activity in the targeted brain region compared to the pre-intervention. **(B)** Block-averaged O_2_Hb amplitude of pre-and post-intervention. The orange line represents the linear O_2_Hb activation of the pre-intervention from the 13 participants. The blue line represents the post-intervention in the same manner. The shaded area (0–10 and 40–50 s) represents the resting state (fixation phase), and the white area (10–40 s) indicates activation during the two-back task. **(C)** Average change of GLM value from the 13 participants after the 4-week training. Only the mean value of GLM during the task was compared (pre: *M* = 0.040, SE = 0.0067; post: *M* = 0.0059, SE = 0.0038), and a significant decrease was observed (*paired-t*_[12]_ = –2.60; *p* = 0.023). dlPFC, dorsolateral prefrontal cortex; O_2_Hb, oxyhemoglobin concentration; s, second; GLM, general linear model; M, mean; SEM, standard error of the mean.

Compared with post-intervention, no significant differences were observed in the participants’ resting state (i.e., fixation phase), measured before or after the two-back task (Figure 2B, shaded area). The interpolated brain map presented in Figure 2 shows the paired *t*-test result after the 4-week intervention for all 13 participants, revealing that only the mean GLM value during the two-back task differed significantly (Figure 2A; *paired*-*t*_[12]_ = −2.60; *p* = 0.023, Bonferroni-corrected for multiple testing), and the block-averaged O_2_Hb amplitude was reduced during the post-intervention task (Figure 2B).

### 3.3. Neurofeedback data

**FIGURE 3:**
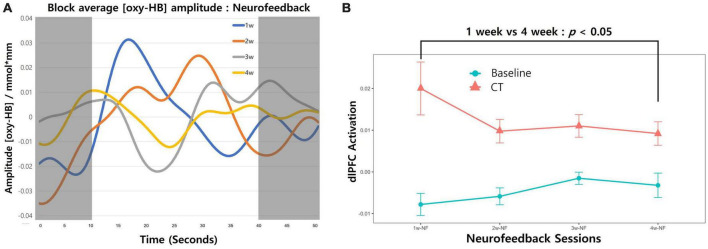
Changes in neurofeedback-induced brain activation. **(A)** The block-averaged O_2_Hb amplitude during the combined cognitive training and neurofeedback intervention is shown. The red line depicts O_2_Hb activation from the first week of intervention for all participants, with the orange, gray, and yellow lines representing the second, third, and fourth weeks, respectively. The shaded regions (0–10 and 40–50 s) correspond to the resting state during the fixation phase, whereas the white area (10–40 s) indicates neural activation occurring during the cognitive training period. **(B)** Neurofeedback-induced activation across 4-week sessions trend lines is presented. Redline with dots represent the averaged O_2_Hb values during the CT. The fixation phase was used for baseline and presented as blueline with dots. Error bars indicate the standard error of the mean (SEM). Comparing the first session and last session change in left-dlPFC revealed a significant decrease (*paired*-*t* [12] = –2.48; *p* = 0.014).

We also calculated brain activation induced by neurofeedback for each session (Figure 3). As our study did not have a control group, a repeated-measures one-way analysis of variance was conducted (group × time) to evaluate training success with averaged neural activation in the targeted brain area. Neurofeedback signals were reduced over time, but the difference was not statistically significant (Figure 3A; *F*_[3,48]_ = 0.971; *p* = 0.414). The induced activation change in the targeted area was obtained only in *the last session* vs. *the first session* (Figure 3B; *paired*-*t*_[12]_ = –2.48; *p* = 0.014).

### 3.4. Correlation analysis

**FIGURE 4:**
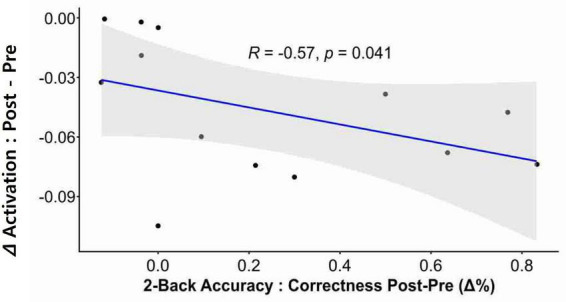
Correlation between neural activation and cognitive performance. A significant correlation was observed between neurofeedback-induced activation and post-intervention n-back performance. The delta (Δ) activation was calculated by subtracting the Pre intervention value from the Post intervention value, where these values refer to the beta-coefficients derived from the GLM. The change in two-back accuracy post-intervention was depicted as delta % (Δ%).

Correlation analysis revealed that neurofeedback-induced activation changes (i.e., reduction) in the left dlPFC were significantly correlated with post-intervention two-back accuracy in the participants (Figure 4; *r* = −0.57, *p* = 0.041).

## 4. Discussion

In this preliminary study, we sought to investigate whether providing a 4-week structured computer-based CT through VR in parallel with fNIRS neurofeedback can aid elderly patients with MCI. We considered specific brain regions to provide feedback on their brain activity to enhance training effectiveness. O_2_Hb in the left-dlPFC was measured during the training to be used as an objective indicator to evaluate efficacy. Based on these results, we obtained two main findings. First, the activity of the targeted brain region increased when the participants first engaged in the training, and after 4 weeks of training, the result showed significantly decreased oxygen saturation in the left dlPFC compared with pre-intervention. Additionally, the neurofeedback signal decreased in the targeted brain area across the CT neurofeedback sessions. Second, the participants showed significantly improved working memory compared with pre-intervention, and the decreased O_2_Hb signal was significantly correlated with improved cognitive performance.

We found evidence from several neuroimaging studies that reported changes in activity in the PFC during or after cognitive tasks. For example, Fishburn et al. (2014) examined the changes in the fNIRS signal in response to an n-back task, and observed increased brain activation as working memory load increased from one-back to three-back trials on the task. They demonstrated that neural changes associated with the modulation of cognitive load and load-dependent activation increased linearly in the left dlPFC. The change in PFC activity during cognitive tasks according to the induced workload has been consistently reported in both laboratory and real-life settings (Ayaz et al., 2012; Herff et al., 2013; Causse et al., 2017; Midha et al., 2021). The current results are consistent with those of the previous research, which reported that higher demands on attention and working memory load would increase O_2_Hb saturation when the participants met the task for the first time (pre-intervention) and CT-neurofeedback for the first week. Of note, the properties of fNIRS were used to measure participants’ workload in the cognitive rehabilitation field to understand the training response.

In addition, we found decreased task-based signals compared with pre-intervention, which is considered to reflect the phenomenon of training effects. Although this study is insufficient to estimate the effectiveness of training because it lacked a control group, the current results partially support the prior observations underlying a decrease in neural activity, which was linked to increased efficiency of maintaining task-related information. For example, Jansma et al. (2001) reported that the blood-oxygen-level-dependent signal of the left dlPFC decreased after the consistent practice of a verbal working memory task, and it was significantly correlated with improved response time and accuracy of the task. Schneiders et al. (2011) reported a significant decrease in the blood-oxygen-level-dependent signal in the right superior, middle frontal gyrus after visual and auditory working memory training. They additionally reported that decreased activation of the right middle frontal gyrus was specifically associated with visual working memory training, which increased training gain, as opposed to auditory working memory training.

Moreover, a decrease in PFC activity was reported in a task-based fNIRS study (Hosseini et al., 2016) that conducted fNIRS neurofeedback in parallel with computerized CT in 20 healthy adult participants. The results showed improved executive function performance, including working memory measures after four training sessions, significantly correlated with reduced training-related O_2_Hb signals in the PFC. To address the mechanism of neural efficiency via training, Curtin et al. (2019) monitored the brain activity of 16 healthy adults using fNIRS during CT. They used excitatory repetitive transcranial magnetic stimulation to investigate whether neuromodulation can enhance neural efficiency and training. The authors demonstrated that a decrease in activity in the left dlPFC was significantly correlated with improved task performance due to practice, and high-frequency repetitive transcranial magnetic stimulation in the left dlPFC, which was provided before the cognitive task, led to a decrease in oxygenation level and an increase in task efficiency compared with the SHAM group. Overall, these domain-specific phenomena indicate that the brain becomes more efficient at performing tasks that are trained, and as a result, less mental effort is required to complete them (Kelly and Garavan, 2005; Qi and Constantinidis, 2013). A decrease in PFC activity after practice might be used to evaluate the training effect or set an individualized difficulty level.

Lastly, the results of the current study show that the participants’ working memory performance after the 4-week training was significantly improved compared with the pre-intervention. Previous studies on working memory training have shown that this training improves multiple cognitive functions in patients with MCI (Finn and McDonald, 2014; Hyer et al., 2016; Zhang et al., 2019). In this study, the provided CT was relatively short-term compared to the previous study, with 20 min of training once a week, four times in total. Our results may reflect the effect of the neurofeedback training design, which self-evaluates activity in the targeted brain region while conducting CT.

Despite differences in spatial resolution and instrumentation, fNIRS and fMRI can measure the hemodynamic response in the cerebral cortex evoked by a particular task. These methods are believed to measure the same underlying biological signals (Huppert et al., 2006), and our results correspond with those obtained using fMRI. These findings suggest that fNIRS and fMRI can be used interchangeably to study task-evoked hemodynamic responses in the brain.

This preliminary study showed the possibility of using VR + CT neurofeedback to improve cognitive function in elderly patients with MCI. However, this study had several limitations that should be considered in future research. First, we did not have a control or SHAM group to measure the effects of neurofeedback training. During the study period, the COVID-19 pandemic situation in Republic of Korea was at its peak, and it was challenging to recruit participants due to the vulnerability of the elderly. Further research should aim to incorporate a more diversified experimental design, potentially including groups matched for age and IQ, a SHAM feedback group, and comparison groups receiving different treatments. Also, considering that MCI is not solely attributed to neurodegenerative diseases, a thorough characterization of group composition is essential during the MCI screening process. This approach would enable a clearer elucidation of the main effects of CT and neurofeedback. Second, as our NIRS system could not measure the whole brain, we could not investigate changes in activity in various brain regions according to the progress of CT neurofeedback. Recent studies have shown that the pathobiological characteristics of degenerative disorders are highly associated with salient networks such as the frontoparietal, central executive, and default mode networks (Iturria-Medina and Evans, 2015; Benson et al., 2018). Additional studies investigating more specific brain regions underlying the mechanisms of MCI are needed. Third, we did not follow-up with the participants to investigate the long-term effects of the training. It is recommended that future neurofeedback studies should include follow-up assessments to investigate the long-term stability of training effects, and there is a need for more well-controlled studies to determine the specific conditions under which neurofeedback can lead to stable improvements in cognitive and clinical outcomes (Enriquez-Geppert et al., 2017). Therefore, future studies should test the long-term effects of CT neurofeedback. Lastly, we acknowledge our limited sample size as a constraint in this preliminary study. Given the very small sample and the reported significant findings, it is important to note that these may be overestimated (Algermissen and Mehler, 2018). To strengthen statistical power and external validity, future research should consider with a larger participant sample.

To address the stated limitations and effectively structure fNIRS studies, endorse the use of preregistration, as recently detailed by Schroeder et al. (2023). This guide, tailored to fNIRS research, offers key design principles and comprehensive study planning recommendations. As fNIRS research evolves, the adoption of these robust methodologies will enhance transparency and the credibility of findings.

## 5. Conclusion

In conclusion, this study demonstrated that fNIRS could detect cognitive load, rendering it a valuable tool in cognitive rehabilitation. This technique can be used to understand the mental effort of individuals with MCI and may also have the potential for use in evaluating brain-computer interfaces. Additionally, providing real-time feedback based on objective indicators may help increase neural efficiency, as participants are motivated and engaged when monitoring their brain activity. These findings highlight the potential of fNIRS as an effective tool for understanding and improving cognitive functions.

## Data availability statement

The original contributions presented in this study are included in this article/Supplementary material, further inquiries can be directed to the corresponding author.

## Ethics statement

The studies involving human participants were reviewed and approved by the Dankook University Hospital Review Board. The patients/participants provided their written informed consent to participate in this study.

## Author contributions

IL, DK, and JL contributed to conception and experimental design of the study. IL and DK organized the database. SK and HK developed virtual reality software and cognitive training protocol. DK contributed to recruitment and the participants training progress. IL, DK, and UC performed the statistical analysis. IL wrote the first draft of the manuscript. DK, SK, HK, UC, and JL wrote sections of the manuscript. All authors contributed to manuscript revision, read, and approved the submitted version.
