# Supplementary figures and images for: Cognitive training based on functional near-infrared spectroscopy neurofeedback for the elderly with mild cognitive impairment: a preliminary study
Source: Front Aging Neurosci. 2023 Jul 26;15:1168815. doi: 10.3389/fnagi.2023.1168815 (PMC10410268; doi:10.3389/fnagi.2023.1168815)

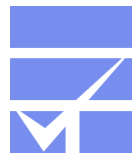

# CONSORT

TRANSPARENT REPORTING of TRIALS

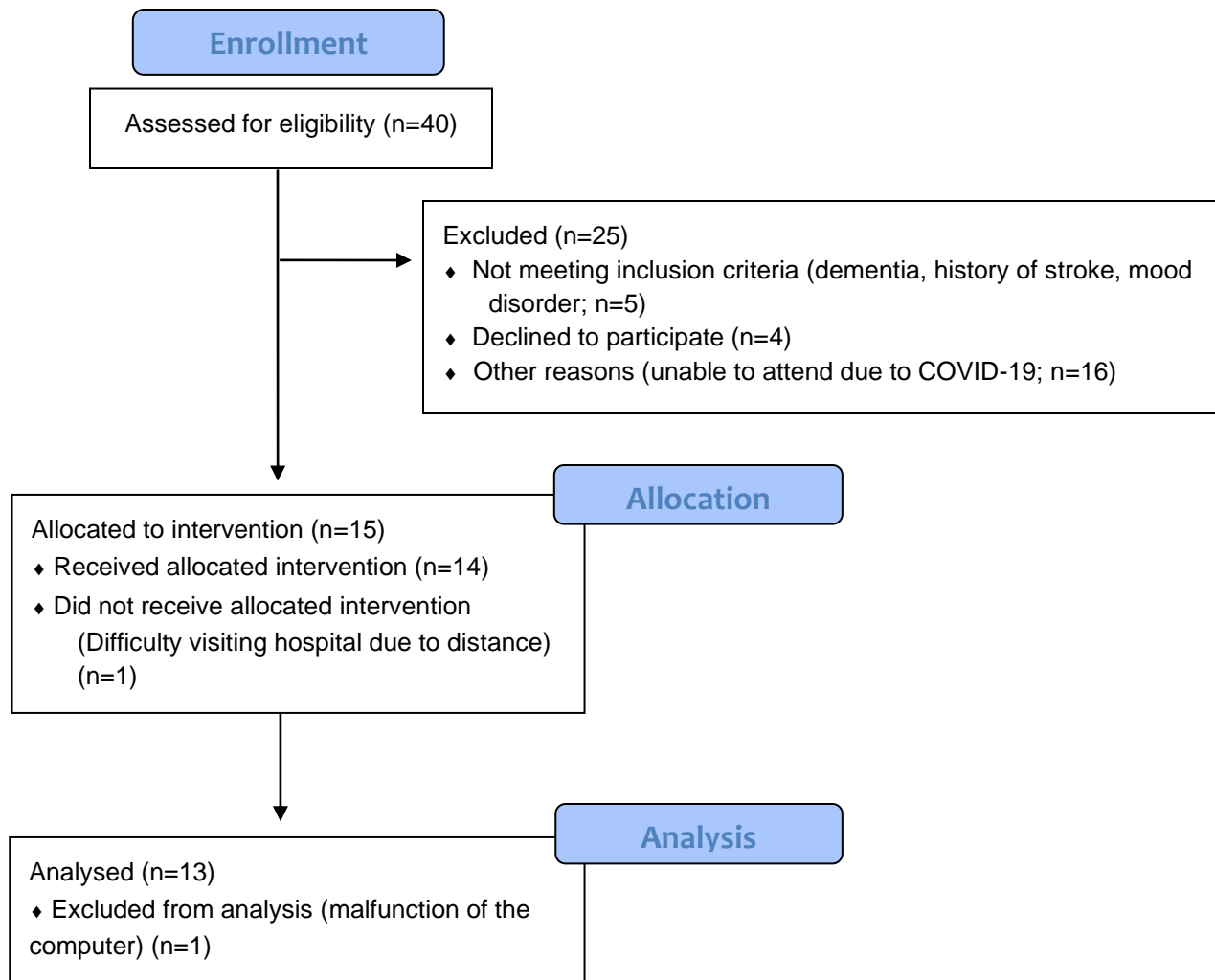

Supplement: Supplementary file 1 [file Data_Sheet_1.PDF]
