# Supplementary material for: Cognitive training based on functional near-infrared spectroscopy neurofeedback for the elderly with mild cognitive impairment: a preliminary study
Source: Front Aging Neurosci. 2023 Jul 26;15:1168815. doi: 10.3389/fnagi.2023.1168815 (PMC10410268; doi:10.3389/fnagi.2023.1168815)
Supplement: Supplementary file 2 [file Data_Sheet_2.pdf]

# CRED-nf checklist summary

*26 June, 2023*

**Manuscript title:** Cognitive training based on functional near-infrared spectroscopy neurofeedback for the elderly with mild cognitive impairment: A preliminary study
**Corresponding Author:** Jung Jae Lee
**Corresponding author email:** mdjjlee@dankook.ac.kr

| | **Checklist item** | **Manuscript Details** |
|---|---|---|
| **Item No.** | | |
| **Pre-experiment** | | |
| 1a | Pre-register experimental protocol and planned analyses | *This experiment was not preregistered* |
| 1b | Justify sample size | 2.1 Participants. In this preliminary study, our heuristic approach guided us to aim for a minimum of 40 participants, establishing a foundational dataset for our analysis (Lakens, 2022). However, our study faced the unforeseen challenge of participant dropout due to the COVID-19 pandemic mandated that we proceed with our research using a diminished final sample size for our analysis. Fifteen elderly patients with MCI (12 females, mean [standard deviation (SD)] age = 70.92 [5.86]) participated in this study. One patients refused to participate after the baseline measurement, and one patient's data could not be used for the study results owing to the malfunction of the NIRS device. Finally, data from 13 patients were analyzed (Provided in the format of a CONSORT diagram). |
| 2a | Employ control group(s) or control condition(s) | 2.6 Statistical Analysis. As our study did not include a SHAM or control group, we used a paired ttest to evaluate the statistical significance of pre-post changes in neuropsychological assessments, behavioral performance, and neural activation (i.e., O2Hb) in the two-back task. Bonferroni corrections to control family-wise error rates were used to assess statistical significance in the context of multiple tests. In cases where the assumption of normality was violated, we used twotailed Mann–Whitney U or Wilcoxon signed-rank tests. |

| | | |
|---|---|---|
| 2b | When leveraging experimental designs where a double-blind is possible, use a double-blind | *NA: A double-blind was not appropriate for this experiment* |
| 2c | Blind those who rate the outcomes | *Those who rated the outcome were not blind to group assignment* |
| | Blind those who analyse the data | *Those who analysed the data were not blind to group assignment* |
| 2d | Examine to what extent participants and experimenters remain blinded | *No measures were taken to examine whether participants and experimenters remained blind* |

| | | |
|---|---|---|
| 2e | In clinical efficacy studies, employ a standard-ofcare intervention group as a benchmark for improvement | *NA: This is not a clinical efficacy study* |
| 3a | Collect data on psychosocial factors | The following results are based on the final participant set consisting of 13 participants in the experiment. They successfully underwent baseline and post-intervention evaluations and completed all VR + CT neurofeedback training sessions. Participants' mean age was 70.92 years (SD = 5.86), and there was only one male participant. |
| 3b | Report whether participants were provided with a strategy | *The manuscript does not report whether strategies were provided* |
| 3c | Report the strategies participants used | *The strategies participants used were not recorded or not reported in the manuscript* |

| | | |
|---|---|---|
| 3d | Report methods used for online-data processing and artifact correction | Structured preprocessing for fNIRS data was performed to analyze pre-post n-back task-based NIRS data and NIRS-neurofeedback training data. The MATLAB toolbox, NIRS-KIT, was used for data preparation, quality control, preprocessing, and statistical analyses (Hou et al., 2021). Raw signals were bandpass-filtered between 0.01 and 0.08 Hz using a third-order IIR Butterworth filter to eliminate physiological artifacts. The NIRS-KIT also provides motion correction to effectively remove both spike artifacts and baseline shifts, and temporal derivative distribution repair was chosen (Fishburn et al., 2019). Next, an individual-level analysis was conducted to obtain task-related neural activity based on the general linear model (GLM). The estimation of the GLM parameters from the task variables was used to calculate the weight coefficient in the linear model after inputting the user-defined contrast vectors with condition-wise effects of interest. The block average amplitude value can be estimated using the above procedure for the group-level analysis. Finally, we used the EasyTopo toolbox, which provides interpolated three-dimensional visualization on a standard brain surface to present three-dimensional visualization (Tian et al., 2013). The integrated statistical values and the Montreal Neurological Institute and |
| | | Hospital coordinates of each channel were calculated, and all colors of the brain surface represented the t-statistic values of the group-level t-test. |
| 3e | Report condition and group effects for artifacts | *Condition and group effects for artifacts were not measured, or not reported in the manuscript* |

| | | |
|---|---|---|
| 4a | Report how the online-feature extraction was defined | In the procedure of the cognitive training combined with neurofeedback, the image set was presented to the participant for 6 s as the encoding phase in each trial. A delay period of 10 s was used as the retention phase, following a single image presented on the VR screen. In the inquiry phase, the participant had to respond within 4 s if the image was included in the original stimuli set. The fixation period was 8 s to separate the phase from the subsequent trial. Each training session consisted of 20 trials, and the participants underwent once-weekly training sessions for four weeks. We conducted a delayed working memory task to proceed with the neurofeedback training. Reflecting on the characteristics of older adults in that they may not have known letters, we provided images that can be encountered in daily life as memory tasks. In each trial, a set of five varied images was presented, chosen randomly from a pool of 80 unique images representing a wide range of everyday items, from animals to household goods to clothing. Once an image was correctly identified during a trial, it was removed from the selection pool for subsequent trials, ensuring that the stimuli remained fresh and engaging for the participants throughout the neurofeedback training. |
| 4b | Report and justify the reinforcement schedule | In each trial, the participants were presented with feedback regarding brain activity in the leftdlPFC and their behavioral performance. The NIRS signal was recorded during the trial (i.e., fixation, encoding, and retention phases), and OHb measurements over a 24-s window were used for neurofeedback. |
| 4c | Report the feedback modality and content | The feedback was visually presented as a bar plot that showed changes in neural activity during the previous trial. Absolute OHb values were calculated to represent decreased or increased activity in the targeted channel. |
| 4d | Collect and report all brain activity variable(s) and/or contrasts used for feedback, as displayed to experimental participants | *All brain activity variable(s) and/or contrasts used for feedback, as displayed to experimental participants were not collected or are not reported in the manuscript* |

| | | |
|---|---|---|
| 4e | Report the hardware and software used | fNIRS records changes in oxygenated (O2Hb) and deoxygenated (HHb) hemoglobin relative to a baseline, and the amount of local O2Hb infers the amount of local brain activation via the process of hemodynamic coupling, wherein increases in cortical activation lead to increases in O2Hb and decrease in HHb (Haeussinger et al., 2014). While most commercial NIRS systems only measure changes from a baseline, our study utilized the ISS OxiplexTS (ISS, USA), a commercially available frequency-domain NIRS (fdNIRS) system that can calculate absolute hemoglobin concentrations (Fantini et al., 1999), that is, it has a different mechanism from continuous-wavebased fNIRS. The probe includes one detector and four fiber optic sources, emitting light at 690 and 830 nm into the tissue with a modulated frequency of 110 MHz. After a 30-minute warm-up period and calibration with phantoms of known absorption and scattering coefficients, the NIRS probe was placed on the right forehead as close to the hairline as possible and secured with Velcro straps. If we keep this procedure, we can measure the absolute hemoglobin concentrations without short distance. The frequency domain-multiple distance NIRS (ISS, USA) was used for pre-and post-tests and neurofeedback sessions. The optode sensor featured a flexible rubber molded construction w |
| | | ith four emitter positions with emitter-detector distances of 2.0, 2.5, 3.0, and 3.5 cm. The optode sensor was placed at the center with the innermost channel of the international 10–20 electroencephalogram system (Jasper, 1958). |
| 5a | Report neurofeedback regulation success based on the feedback signal | Compared with post-intervention, no significant differences were observed in the participants' resting state (i.e., fixation phase), measured before or after the two-back task (Figure 1b, shaded area). The interpolated brain map presented in Fig. 1 shows the paired t-test result after the 4-week intervention for all 13 participants, revealing that only the mean GLM value during the two-back task differed significantly (Figure 1a; paired-t[12] = –2.60; p = 0.023, Bonferroni-corrected for multiple testing), and the block-averaged O2Hb amplitude was reduced during the post-intervention task (Figure 1b). Figure 1. Changes in leftdlPFC activity after VR+CT-neurofeedback. a) Difference between pre-and post-intervention in left-dlPFC activity in response to the training: Contrasted t-map shows significantly decreased activity in the targeted brain region compared to the pre-intervention. b) Block-averaged O2Hb amplitude of pre-and post-intervention. The orange line represents the linear O2Hb activation of the pre-intervention from the 13 participants. The blue line represents the post-intervention in the same manner. The shaded area (0–10 s; 40–50 s) represents the resting state (fixation phase), and the white area (10–40 s) indicates activation during the two-back task. c) Average change of GLM value from the 13 p |

| | | |
|---|---|---|
| | | articipants after the 4-week training. Only the mean value of GLM during the task was compared (pre: M = 0.040, SE = 0.0067; post: M = 0.0059, SE = 0.0038), and a significant decrease was observed (paired-t[12] = –2.60; p = 0.023). |
| 5b | Plot within-session and between-session regulation blocks of feedback variable(s), as well as pre-to-post resting baselines or contrasts | Figure 2. Changes in neurofeedback-induced brain activation. a) The block-averaged O2Hb amplitude during the combined cognitive training and neurofeedback intervention is shown. The red line depicts O2Hb activation from the first week of intervention for all participants, with the orange, gray, and yellow lines representing the second, third, and fourth weeks, respectively. The shaded regions (0–10 s; 40–50 s) correspond to the resting state during the fixation phase, whereas the white area (10–40 s) indicates neural activation occurring during the cognitive training period. b) Neurofeedback-induced activation across 4-week sessions trend lines is presented. Redline with dots represent the averaged O2Hb values during the CT The fixation phase was used for baseline and presented as blueline with dots. Error bars indicate the standard error of the mean (SEM). Comparing the first session and last session change in left-dlPFC revealed a significant decrease (paired-t [12] = –2.48; p = 0.014). |
| 5c | Statistically compare the experimental condition/group to the control condition(s)/group(s) (not only each group to baseline measures) | *The manuscript does not statistically compare the experimental condition/group to the control condition(s)/group(s)* |
| 6a | Include measures of clinical or behavioural significance, defined a priori, and describe whether they were reached | Significant improvement in the working memory scores was observed after the 4-week training compared to pre-intervention : the Boston naming test (paired-t[12] = 2.22; p = 0.023), word list recognition (paired-t[12] = 2.12; p = 0.027), constructional recall (paired-t[12] = 2.29; p = 0.020), and Digit Span Test (forward: paired-t[12] = 2.01; p = 0.032, total: paired-t[12] = 3.24; p = 0.003). There was a similar improvement in the working memory task: the two-back task was performed accurately (paired-t[12] = 2.25; p = 0.021). |
| 6b | Run correlational analyses between regulation success and behavioural outcomes | Correlation analysis revealed that neurofeedback-induced activation changes (i.e., reduction) in the left dlPFC were significantly correlated with post-intervention two-back accuracy in the participants (Fig. 3; r = –0.57, p = 0.041). |

**Data storage**

| | | |
|---|---|---|
| 7a | Upload all materials, analysis scripts, code, and raw data used for analyses, as well as final values, to an open access data repository, when feasible | *No additional documents related to the materials, analysis scripts, code, raw data, or final values are available for this manuscript* |
